# Partnerships in mental healthcare service delivery in low-resource settings: developing an innovative network in rural Nepal

**DOI:** 10.1186/s12992-016-0226-0

**Published:** 2017-01-13

**Authors:** Bibhav Acharya, Duncan Maru, Ryan Schwarz, David Citrin, Jasmine Tenpa, Soniya Hirachan, Madhur Basnet, Poshan Thapa, Sikhar Swar, Scott Halliday, Brandon Kohrt, Nagendra P. Luitel, Erick Hung, Bikash Gauchan, Rajeev Pokharel, Maria Ekstrand

**Affiliations:** 1 Bayalpata Hospital, Possible, Sanfebagar-10, Achham Nepal; 2 Department of Psychiatry, University of California, San Francisco, 401 Parnassus Ave, Langley Porter, San Francisco, CA 94143 USA; 3 Shared Minds, Boston, MA USA; 4 Department of Medicine, Division of Global Health Equity, Brigham and Women’s Hospital, Boston, MA USA; 5 Department of Medicine, Division of General Pediatrics, Boston Children’s Hospital, Boston, MA USA; 6 Department of Medicine, Harvard Medical School, Boston, MA USA; 7 Department of Medicine, Division of General Internal Medicine, Massachusetts General Hospital, Boston, MA USA; 8 Department of Anthropology, University of Washington, Seattle, WA USA; 9 Department of Global Health, University of Washington, Seattle, WA USA; 10 Henry M. Jackson School of International Studies, University of Washington, Seattle, WA USA; 11 Department of Psychiatry, University of Minnesota, Minneapolis, MN USA; 12 Faculty of Medicine, Department of Psychiatry, B.P. Koirala Institute of Health Sciences, Dharan, Sunsari Nepal; 13 Psychiatric Department, Kathmandu Medical College, Kathmandu, Nepal; 14 Research Department, Transcultural Psychosocial Organization Nepal, Kathmandu, Nepal; 15 Department of Psychiatry and Behavioral Sciences, Duke University School of Medicine, Durham, NC USA; 16 Global Health Institute, Duke University, Durham, NC USA; 17 Department of Cultural Anthropology, Duke University, Durham, NC USA; 18 Policy Planning & International Cooperation Division, Ministry of Health, Kathmandu, Nepal; 19 Department of Medicine, University of California, San Francisco, San Francisco, CA USA

**Keywords:** Mental health, Global health, Nepal, Partnerships, Low— and middle-income countries

## Abstract

**Background:**

Mental illnesses are the largest contributors to the global burden of non-communicable diseases. However, there is extremely limited access to high quality, culturally-sensitive, and contextually-appropriate mental healthcare services. This situation persists despite the availability of interventions with proven efficacy to improve patient outcomes. A partnerships network is necessary for successful program adaptation and implementation.

**Partnerships network:**

We describe our partnerships network as a case example that addresses challenges in delivering mental healthcare and which can serve as a model for similar settings. Our perspectives are informed from integrating mental healthcare services within a rural public hospital in Nepal. Our approach includes training and supervising generalist health workers by off-site psychiatrists. This is made possible by complementing the strengths and weaknesses of the various groups involved: the public sector, a non-profit organization that provides general healthcare services and one that specializes in mental health, a community advisory board, academic centers in high- and low-income countries, and bicultural professionals from the diaspora community.

**Conclusions:**

We propose a partnerships model to assist implementation of promising programs to expand access to mental healthcare in low- resource settings. We describe the success and limitations of our current partners in a mental health program in rural Nepal.

## Background

The large burden of mental illness in low— and middle-income countries (LMICs) [1] has demonstrated a great need for identifying, testing, and scaling successful mental healthcare interventions [2–4]. Efforts are underway to adapt and scale-up successful programs [5–7]. The process of replicating such programs in new settings faces challenges inherent to the practice of global mental health: cultural factors that are unique to the new community, limitations to financial and human resources outside of funded research studies, absence of supportive national mental health policies, and a lack of robust healthcare delivery systems capable of integrating mental healthcare services [1]. Attempts to implement services that fail to address these complex determinants of health can result in poor, fragmented care and cause harm to patients [8, 9].

Although the World Health Organization (WHO) has developed a comprehensive action plan to guide mental healthcare service delivery [10], the process of adapting such programs requires diverse partners with complementary skills. Virtually all examples of partnerships in the literature are between specific institutions in high-income countries (HICs) and LMICs [11], including partnerships specifically in mental health [12–14]. Such models often focus on the experience of research, training, or capacity building of a specific LMIC institution. However, a model that not only incorporates HIC institutions but also mobilizes multiple LMIC-based local stakeholders to deliver mental healthcare services does not exist.

Facing these challenges, our team has engaged with several partners to deliver mental healthcare services in rural Nepal, a low-income country where the per capita gross national income is 718USD [15]. Although over 80% of Nepal’s 30 million people live in rural regions [16], the approximately 50 psychiatrists in the country are clustered largely in the major cities [17]. Our intervention focuses on Achham, one of the poorest districts in Nepal, 12 h from a commercial airport and 14 h from the nearest psychiatrist. Based on human development estimates, Achham ranks 73rd out of 75 districts in Nepal [15]. It was severely affected by the ten year Maoist War that ended in 2006 [18]. Our mental healthcare services are based at Bayalpata Hospital, a district-level government facility in Achham. Since 2008, *Possible*, a non-profit healthcare organization, has been operating Bayalpata Hospital in partnership with the Ministry of Health (MOH). The 25-bed general hospital employs over 150 staff and has seen more than 350,000 patients since opening. We utilize the Collaborative Care Model for mental healthcare delivery [19], and have implemented the following steps: 1) training all generalist clinicians in screening, diagnosing, and treating mental illness; 2) recruiting counselors to provide basic psychotherapy and care coordination; and 3) engaging an off-site psychiatrist to provide supervision and quality control via weekly case review. This strategy for delivering mental healthcare services has proven successful in numerous settings [20]. Here, we describe our partnerships as a case example and a potential model for implementing such a program in similar, low-resource settings.

### The Nepal partnerships network

A successful partnerships model to deliver high quality, culturally-sensitive, and contextually-appropriate mental healthcare services will include stakeholders with complementary skills and limitations. The partnerships we have developed were a result of an iterative process that began when *Possible* and the MOH, who were providing general healthcare services in rural Nepal, became interested in adding mental healthcare services. They sought support from organizations that have specific expertise in mental health and from bi-cultural professionals who could adapt successful programs and train the clinical staff to deliver services. This led to partnerships with local and international academic medical centers who were interested in supporting research, education, and capacity building. In parallel, *Possible* developed a healthcare service users group. The partners communicate at varying frequencies and the primary coordinating body is *Possible* in Achham, where the healthcare delivery system is based. We list the type of partners, explore their strengths and limitations, and describe their specific contributions in our program. The model is summarized in Table 1.

**Table 1:** Contributions and potential limitations of members of the partnership model for a global mental health program

Partner type	Contributions	Potential Limitations
Public Sector Institutions	• Guarantor of health as a right for all citizens. • Able to scale- up and sustain promising programs. • Develop mental health policy.	• May avoid taking risks with new models needed to innovate in healthcare delivery. • May lack capital investment needed for high quality services.
Non-Governmental Healthcare Delivery Organizations	• Invest in innovative projects and take risks with new models. • Clinical and community-based infrastructure allows for integration of mental health into general healthcare services.	• May lack specialized knowledge in vertical programs like mental healthcare. • May harbor stigma against mental health.
Mental Health Organizations	• Specialized focus on cross-cultural adaptation of psychiatric concepts, research scales, and protocols. • Training of health workers. • Advocacy for mental healthcare services.	• May lack local contextual and cultural perspectives of the specific intervention site. • May not have a robust general healthcare delivery infrastructure.
Healthcare Service Users	• Provide feedback and guidance for mental healthcare services. • Advocate for quality services and human rights protections. • Provide local accountability in settings of poor regulatory oversight of services.	• May have limited engagement due to societal stigma. • May not have access to specialized, clinical knowledge. • May lack agency to challenge such established institutions.
Bicultural Professionals	• Provide contextual and culturally-relevant framework for interventions. • Develop clinical protocols in the local language that synthesize evidence and local cultural considerations.	• May not have local presence to provide ongoing training and supervision.
Academic Medical Centers	• Research infrastructure for implementation science, impact evaluation, and structured curriculum development. • Training and mentorship. • Support for principal investigators. • Cross-disciplinary collaborations. • Contextual expertise in healthcare delivery.	• May not have healthcare delivery systems to test interventions in community settings. • May not have local expertise in community settings.

#### Public sector institutions

The public sector is the ultimate guarantor of health as a right for all citizens [21]. The government has the capacity to develop and implement national policies, scale-up promising models of care for long-term implementation, and coordinate with international players to integrate regional and global efforts. An intervention that does not incorporate the public sector will create a parallel structure that inevitably weakens an already fragile national system [22]. An important exception is when the state is hostile towards the very citizens it is mandated to serve and engages in direct or structural violence [23]. Even in such situations, partnerships with certain divisions of the governing body may be forged to ensure access to health in the midst of state-supported humanitarian crises. The limitations of working with the public sector in such settings notwithstanding, we include the public sector as a key partner in most communities in the world.

On average, low-income countries invest 25USD per capita on healthcare [24]. Given this reality, the public sector in low-resource settings may be struggling to meet immediate healthcare demand and could deprioritize investment in high-risk, high-return innovations that may be needed to improve healthcare delivery. Partnering with non-public sector actors can provide an opportunity to co-invest in such models, which, if successful, can be scaled up to the national level. A central challenge in achieving co-investment is what we have experienced as the perceived unwillingness of the public sector to partner with small non-governmental organizations (NGOs). This is often the case because many NGOs are ephemeral and donor-driven, frequently operate with minimal accountability, and compete rather than collaborate with the public sector [22]. NGOs that demonstrate the capacity, track record, and commitment to working together towards national health goals can and have successfully partnered with the public sector.

The MOH invests about 16USD per capita in healthcare, and faces the limitations described above [24]. It has been an early partner for our work in Nepal through a public-private partnership. The MOH develops such partnerships with trusted NGOs to expand its capacity in providing healthcare, particularly for populations in remote regions. Bayalpata Hospital, the hub of the program we describe, is owned by the government. The MOH supplies medications, provides co-financing, and ensures accountability and alignment with national health goals.

#### Non-governmental healthcare delivery organizations

To minimize stigma and optimize access, it is preferable to integrate mental health into general healthcare delivery systems [25]. Stigma is a major barrier to seeking mental healthcare, and providing such service in isolation can reduce access [26]. In contrast to the public sector, NGOs engaged in direct healthcare delivery can often be more flexible and take risks in developing and implementing new strategies for such integration. Implementing a novel mental healthcare delivery program with a partner NGO that provides general healthcare allows a more controlled adaptation to the specific setting, and can deliver a proof-of-concept for scaling- up nationally.

Although such NGOs may have the infrastructure for healthcare delivery, they frequently lack the capacity or expertise to deliver mental healthcare services, driven by the lack of mental health specialists [1] and key services [27] resulting in a large treatment gap among people with mental illness [28]. This can lead to persistent neglect of and stigma against mental healthcare due to incorrect beliefs that mental illness cannot be diagnosed or treated in diverse settings, and that even if treatment is possible, it is less important than other pressing needs [29–31]. Many healthcare delivery organizations may be unaware of the immense toll of untreated mental illness on disability [32] and mortality, as exemplified not just by high rates of suicide around the world [33] but also the 10-20-year shortened lifespan for those with severe mental illness [34]. In addition, adherence to regimens for HIV/AIDS, diabetes, and other ailments is poor among people with untreated mental illness [20, 35]. Even NGOs that recognize the importance of mental health may not implement a formal program because they lack the skills to culturally adapt existing protocols [36, 37]. Organizations that specialize in mental health in the particular community can be key partners to help address these limitations.

*Possible* demonstrated capacity to provide healthcare services by operating a primary care clinic in Achham, where prior to its arrival, there were no allopathic physicians in a district with 250,000 people. The organization consistently expressed a commitment to partnering with the public sector, developed professional relationships with members of the government, and supported Nepal’s national programs like vaccination, HIV/AIDS, tuberculosis, and safe motherhood. This strategy, along with advocacy from local constituencies, has led to a public-private partnership that oversees two district-level hospitals.

#### Mental health organization

Mental health-focused NGOs often have specific expertise and experience providing care for people with mental illness in their communities, training and supporting health workers to deliver care and prevent burn-out, advocating for services, reducing stigma, understanding and responding to culturally-specific explanatory models of mental health, and assisting in cross-cultural adaptation of healthcare interventions. Given poor investment in mental health, such organizations are few in number. They are starting to link, however, with networks like the Movement for Global Mental Health [38] and the Mental Health Innovation Network [39]. At the community level, they often operate in isolation because of stigma, poor funding, and a lack of infrastructure for general healthcare delivery. When funding is provided, such resources may be available in the immediate aftermath of a humanitarian crisis rather than sustained support for durable healthcare systems [40]. An additional limitation is that such NGOs may lack specialized knowledge of specific locations and sub-cultures within a diverse country.

Transcultural Psychological Organization (TPO)-Nepal was founded in 2005 to promote psychosocial well-being of vulnerable populations in Nepal. It has conducted cross-cultural validation of psychiatric tools (e.g., Patient Health Questionnaire-9) [41], and implemented and studied packages of care (e.g., interventions for child soldiers and district-level general mental health interventions) [40, 42]. It has partnered with the Nepali government, and NGOs in Nepal and other low-resource countries [7]. TPO’s Nepali manuals, packages of care, and psychiatric tools are used by our mental health program to screen, assess, and track patient outcomes through time. In addition, counselors trained by TPO are recruited by *Possible* to work full-time in Bayalpata Hospital as care coordinators, who are liaisons between patients, generalists, and the psychiatrist.

#### Healthcare service users

Despite widespread calls to integrate service users [6, 10], very few programs are able to develop this partnership in a meaningful manner. Service users can provide guidance to develop user-centered services, ensure access to appropriate treatments including medications and psychosocial interventions, and provide feedback on existing services. In addition, an empowered group can engage in advocacy and ensure accountability of not just healthcare services but also treatment of users by employers, law enforcement groups, the education sector, and the broader society [43]. Such an involvement provides essential counterweight in settings with poor accountability among institutions involved in healthcare delivery.

Lack of a strong service users group is an important limitation in our program in Nepal. Although there are advocacy groups that work at the national level, we do not have a dedicated mental healthcare service users group in this remote region. *Possible* does have a Community Advisory Board that includes representatives from Bayalpata Hospital’s local community and provides guidance and feedback about the hospital’s work. However, it lacks members who are open about using mental healthcare services. Societal stigma discourages open advocacy, which helps stigma to persist, and this has been a widespread challenge across LMICs [43]. User empowerment can be enhanced by providing services with dignity and full user participation in decisions, promoting self-reliance by helping meet their life goals rather than exclusively focusing on symptom reduction, and supporting incorporation into the family and community [44]. As more treatment and support become available, we expect to invite service users to openly join the advisory board as key partners in the program. In the interim, we receive guidance from international declarations and best practices [45, 46].

#### Bicultural professionals

The migration of clinicians and other professionals from LMICs to HICs is predominantly viewed through the “brain drain” lens [47]. However, as migration continues in the context of globalization and increased mobility for personal and professional reasons, clinicians from the diaspora community can play an important role in navigating cross-cultural differences, translating evidence-based protocols, training health workers in the native language, and facilitating linkages between LMICs and HICs [48, 49]. Their main limitation is that they tend to be based in multiple locations and may not have a reliable presence at the LMIC site to oversee implementation and provide ongoing supervision for generalists. To mitigate this challenge, such professionals can often use their in-country networks to develop international partnerships and to continue to develop in-country capacity.

Shared Minds is a non-profit organization that includes psychiatrists based in Nepal and those from the Nepali diaspora. This group has translated WHO guidelines, mentored HIC-based trainees, and developed lectures in Nepali to train generalist clinicians using culturally-sensitive and contextually-appropriate materials [50]. These lectures are used to train all the clinicians in our program. They also conducted extensive needs assessment among clinicians, which led to a rich understanding of cultural conceptualization and stigma about mental health [48]. These were incorporated into the lectures to present mental healthcare services in a non-stigmatizing manner (e.g., the qualitative study showed that clinicians noted how stigmatizing attitudes about HIV/AIDS had changed, and these themes were incorporated into their mental health training).

#### Academic medical centers

Academic medical centers, based both in HICs and LMICs, can provide human resources and the academic environment to develop and test new models of healthcare delivery. In addition, HIC-based academic medical centers are often able to foster collaborative research across disciplines that may not be as well-developed in LMICs. For instance, expertise in pedagogy and social sciences are critical to health worker training and cross-cultural adaptation of models of care [51]. Conversely, LMIC-based academic medical centers have additional expertise in the context for adapting programs to their specific countries. However, academic medical centers need to partner with healthcare delivery organizations to test interventions in community settings outside of research institutes.

In our program, a psychiatrist (SS) based at Kathmandu Medical College travels every few months to the rural site to provide training and clinical supervision for the generalists. When he is back in Kathmandu, he conducts a weekly case review with health workers in rural Nepal to ensure appropriate quality of care. His institute provides him the flexibility to engage with a remote site, in addition to fostering an academic environment lending contextual expertise in mental healthcare delivery in Nepal.

The University of California, San Francisco has been a key supporter of several academic aspects of our program in Nepal. In addition to supporting a principal investigator (BA) to develop structured curriculum for generalist clinicians in mental health [48], it has fostered collaboration via a global mental health fellowship and implementation science research for the program. This has allowed this mental health program to be situated with the collaborative environment of global health. Harvard Medical School has supported a principal investigator (DM) to conduct implementation science research and has provided a research grant to expand the capacity of the mental health program in the future. The University of Washington has supported two researchers (DC and SHalliday) who implement and study the program and bring expertise from the social sciences. The academic partnerships allow us to go beyond government-mandated metrics (e.g., volume of patients seen) and conduct rigorous evaluations to measure process (e.g., pre- and post-test performance of clinicians who receive mental health training), quality (e.g., rate of antidepressant prescribed at therapeutic dosages), and clinical outcomes (e.g., rate of response in depression, measured by >50% reduction from baseline scores in Patient Health Questionnaire-9 items).

### Limitations

We have not included a singular, dedicated financing mechanism in the model because every situation will warrant a creative approach to pay for healthcare delivery. In our program, payment for services comes from the public sector and funds raised by *Possible* from philanthropic foundations and individual donors, while research is supported by academic centers and their associated funders such as the National Institutes of Health. In other settings, there could be national or local health insurance, dedicated philanthropy, large scale research programs, integration with better-funded programs like HIV/AIDS services, or other creative mechanisms. A model such as this allows for leveraging diverse sources of funding and can help avoid point-of-care user fees, which are a prohibitive barrier for the most vulnerable people [52]. It is also important to note that as other settings utilize different sources of funding, there will be differential influence on the partnerships.

Another important limitation is the exclusion of allied groups like traditional healers, schools, employers, and civil society, all of which are important players in a broad initiative that reaches beyond our clinical program. Similarly, we have not explicitly noted the impact of international organizations like the WHO, given our focus on partners who directly collaborate to deliver healthcare. However, our program utilizes WHO materials like the Mental Health Action Plan and the mhGAP Intervention Guide [10, 36, 37]. Another limitation is that our suggestions, which are based on one LMIC, may not be generalizable to other LMICs. Other countries may lack a mental health policy, have varying levels of coordination between national and local authorities, and have differential health worker capacity. Finally, we have discussed a potential model without evaluation data on its effectiveness. In fact, there is a dearth of robust studies that assess institutional partnerships [11].

Ongoing challenges for our partnerships model include: lack of structured and regular communication among the partners, lack of indicators to assess levels of engagement and ways to optimize it, and the overall lack of resources that prevent the partners from frequent visits to the healthcare delivery site. Such challenges can be overcome by accessing dedicated funding to support formal collaborations like the Collective Impact Framework [53]. It is also important to note that these partnerships specifically focus on facility-based mental healthcare delivery. As such, it is beyond the model’s scope to establish a national mental health program that would include interventions that span preventive services, social services, homes, schools, prisons, and beyond.

## Conclusions

With compelling evidence to increase investment in global mental health [54, 55] and the inclusion of mental health in the Sustainable Development Goals [56], practitioners and national governments are being urged to adapt and scale-up mental healthcare services. Our partnerships were developed organically and iteratively, based on the needs of the healthcare delivery system that was interested in providing high-quality mental healthcare services. Despite having several limitations, these partnerships serve as a case study and potential model to fill a large gap in the literature of practical guides on successful program adaptation and implementation. The partnerships network includes numerous LMIC-based stakeholders and stands in contrast with common partnerships that tend to be built between a single institution in a HIC and one in a LMIC. However, interventions that fail to integrate diverse partners may create a program that has the limitations described above. A comprehensive partnership model can help address such limitations by leveraging resources to build a program that expands access to mental healthcare services, ensures quality care, is culturally sensitive and contextually relevant, and produces scientific knowledge.

## Abbreviations

HIC: High-income country
HIV/AIDS: Human immunodeficiency virus/acquired immune deficiency syndrome
LMICs: Low— and middle-income countries
MOH: Ministry of Health (Nepal)
NGOs: Non-governmental organizations
TPO: Transcultural Psychological Organization
USD: United States dollars
WHO: World Health Organization
